# Possibilities for the efficient utilisation of spent geothermal waters

**DOI:** 10.1007/s11356-014-3076-4

**Published:** 2014-06-07

**Authors:** Barbara Tomaszewska, Andrzej Szczepański

**Affiliations:** 1 Mineral and Energy Economy Research Institute of the Polish Academy of Sciences, Wybickiego 7 31-261 Kraków, Poland; 2 AGH University of Science and Technology, Al. A. Mickiewicza 30 30-059 Kraków, Poland

**Keywords:** Geothermal water, The parameters of Polish geothermal waters, Water treatment, Water mineralisation, Desalination, Water and concentrate used

## Abstract

Waters located at greater depths usually exhibit high mineral content, which necessitates the use of closed systems, i.e. re-injecting them into the formation after recovering the heat. This significantly reduces investment efficiency owing to the need to drill absorption wells and to perform anti-corrosion and anti-clogging procedures. In this paper, possibilities for the efficient utilisation of cooled geothermal waters are considered, particularly with respect to open or mixed geothermal water installations. Where cooled water desalination technologies are used, this allows the water to be demineralised and used to meet local needs (as drinking water and for leisure purposes). The retentate left as a by-product of the process contains valuable ingredients that can be used for balneological and/or leisure purposes. Thus, the technology for desalinating spent geothermal waters with high mineral content allows improved water management on a local scale and makes it possible to minimise the environmental threat resulting from the need to dump these waters into waterways or surface water bodies and/or inject them into the formation. The paper is concerned with Polish geothermal system and provides information about the parameters of Polish geothermal waters.

## Introduction

During the last decade, an increase in global geothermal energy usage has been recorded. Technological development and the increased efficiency of the systems used as well as environmental and economic considerations have played a fundamental role in this process (Bundschuh and Hoinkis 2012; Lund et al. 2010). As at the end 2009, worldwide total installed capacity in systems designed for direct use was 50,583 MWt, and heat consumption was 438,071 TJ (121,696 GWh) (Kępińska 2010). Geothermal energy is used directly (for heating, leisure and balneological purposes, in agriculture and aquaculture where thermophilic species are bred, etc.) in 78 countries of the world (Kępińska 2010). In terms of annual heat consumption, the top five countries that use geothermal energy are China, the USA, Sweden, Turkey and Japan, which together account for 55 % of total annual global geothermal heat consumption (Lund et al. 2010). Electricity from geothermal steam is generated in 24 countries, including the USA, Philippines, Indonesia, Mexico and Italy (Bertani 2010).

In Europe, low-temperature energy resources prevail, i.e. water reservoirs with temperatures below 150 °C. These are mainly present in sedimentary—limestone, dolomite, sandstone—and igneous (crystalline, volcanic) rocks (Kępińska 2010). Geothermal energy is usually used directly but also indirectly—to generate electricity in binary cycle power plants (Austria, Germany).

The presence of geothermal waters that provide potential sources of usable energy depends in general on two basic factors (Szewczyk 2010):

- Geophysical—the Earth geothermal flux and the associated rock mass temperature.
- Hydrogeological—the hydraulic conductivity of aquifers and the mineralisation of underground waters.

About two-thirds of the territory of Poland is considered promising in terms of the technological feasibility of developing its geothermal energy potential (Górecki (2006). Therefore, the interest of Polish municipal local government bodies and businesses in the use of geothermal waters for heating, and also for balneological and leisure purposes, has been growing in recent years (Bujakowski et al. 2010; Bujakowski, 2010; Tomaszewska et al. 2010). Key factors that determine the conditions in which geothermal waters are used, the amount of energy obtained and the manner in which cooled water is utilised include water salinity and the presence of specific ingredients. Elevated salinity levels and the presence of microelements such as boron, barium, strontium, fluorides, bromides and heavy metals may often lead to difficulties related to the utilisation of spent waters. Only a few Polish geothermal facilities operate in a closed system, where the water is injected back into the formation after having been used. Open (with water dumped into surface waterways or sewerage systems) or mixed (only part of the water is re-injected into the formation via absorption wells while the rest is dumped into rivers) arrangements are more frequently used. This issue raises many doubts related to reservoir engineering concerning the sustainability of the system and ensuring proper parameters for its long-time operation, but environmental concerns are present as well, arising from the potential adverse impact of spent waters on receiver quality with respect to surface waterways and water bodies.

In this paper, possibilities for the efficient utilisation of cooled geothermal waters are considered, particularly with respect to open or mixed geothermal water installations.

### Salinity of geothermal waters used

**Fig. 1:**
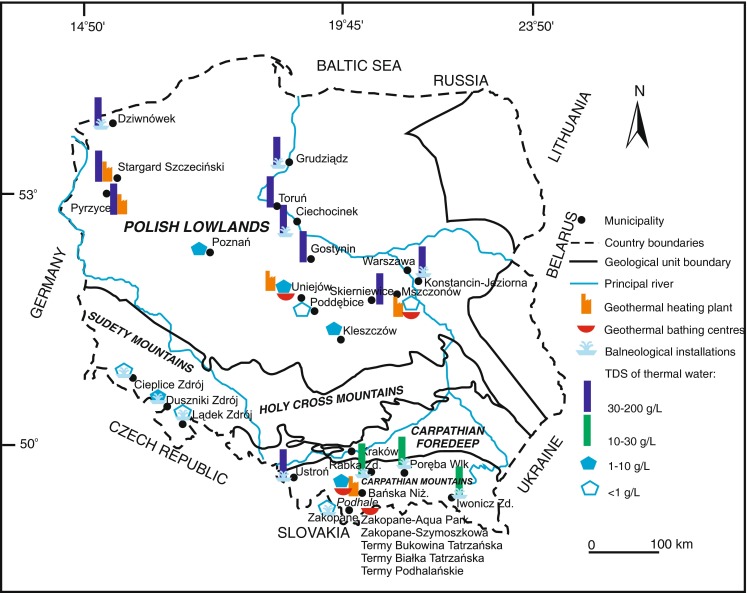
Presence and salinity of thermal waters in Poland

Geothermal energy resources in Poland are associated with groundwater present at depths of up to ca. 3,000–3,500 m within certain regional geological units: the Polish Lowlands, the Carpathians, the Carpathian Depression and the Sudetes (Fig. 1). Relatively favourable geothermal conditions are only present in north-western Poland; in other areas of the country, they are similar to those found in most European countries. According to Szewczyk (2010), temperatures at the depth of 2,000 m range from around 30 °C within anorthosite massifs in north-eastern Poland to more than 92 °C in the central part of the Fore-Sudetic Monocline. High temperatures are present in the lithosphere in the north-western part of Poland, which includes the Szczecin Synclinorium (Szewczyk 2010; Sowiżdżał 2010; Sowiżdżał et al. 2013). This zone can be clearly seen to extend to the north-eastern part of Germany (Szewczyk and Gientka 2009; Szewczyk 2010).

The studies conducted in Poland so far, confirmed by a number of research and investment projects implemented since the early 1980s, have indicated that the use of geothermal waters should be based primarily on the potential accumulated in the Lower Cretaceous and Lower Jurassic reservoir rocks of the Polish Lowlands and the Middle Triassic, Jurassic and Middle Eocene reservoirs of the Podhale geothermal system within the Inner Carpathians (Barbacki and Bujakowski 2004; Bujakowski 2010; Chowaniec 2003, 2009; Górecki 2006; Górecki 2010; Górecki et al. 2010; Kępińska, 2006). The geothermal waters present in the aforementioned geological structures are fresh waters and waters with low mineral content as well as brines with mineral content exceeding 100 g/L (Barbacki and Bujakowski 2004; Bujakowski et al. 2012; Tomaszewska 2011; Kępińska 2006).

In terms of the amount of dissolved substances (water mineralisation), the waters in question can be divided into four groups (Fig. 1): (1) brine waters (highly mineralized waters with a concentration of mineral solutes ranging from 30 to more than 200 g/L), (2) saline waters (waters with dissolved substance content ranging from 10 to 30 g/L), (3) brackish waters (with low mineral content (salt content ranging from 1 to 10 g/L), and (4) fresh waters (with mineral content below 1 g/L). The salinity of geothermal waters present in Poland varies widely. This determines, in effect, the manner in which spent water can be utilised. Brines and saline waters (groups 1 and 2) usually circulate in closed systems (geothermal doublets). Spent water is re-injected into geological formations. This is the best manner of utilising such water from the hydrogeological, hydrodynamic and environmental points of view. Only in balneological facilities are brines dumped into surface waterways or sewerage systems following their dilution and use. The volume of such water that is extracted has so far been limited to at most a few cubic metres per hour. Geothermal waters included in groups 3 and 4, circulate in open or semi-open systems. As a result, significant amounts of geothermal water (from around a dozen to several hundred cubic metres per hour) are usually dumped into surface waterways.

In accordance with the Polish Water Law (2012), used geothermal waters discharged into surface waterways are considered as waste water, and therefore, their quality must meet the requirements of the regulations on the use of water, in particular, the Regulation of the Minister of the Environment concerning the conditions to be met by water discharged into surface waterways or the soil and on substances which are particularly harmful to the aquatic environment (2006). A water permit is necessary to discharge used water into surface waterways.

### Use of waters with high and medium mineral contents

Geothermal waters with high and medium mineral contents are mostly used for heating purposes. Since these waters usually exhibit increased concentrations of chloride ions, sodium, sulfates, calcium and magnesium, iron, iodine, bromides, strontium and fluorine, and sometimes also hydrogen sulfide, they are used for therapeutic and balneological purposes as well. Requirements for bathing water are as follows: the mineral content of waters used for leisure purposes must not exceed 30 g/L (in temperatures ranging from 24 to 30 °C), while for therapeutic purposes it must not exceed 50 g/L (in temperatures ranging from 28 to 42 °C) (Płochniewski, 1990). Therefore, water often has to be diluted in order to be used for therapeutic or balneological purposes. Iodine and bromide salts are also recovered from geothermal waters (Iwonicz-Zdrój and Lubatówka, Ciechocinek). Cosmetics have also been manufactured on the basis of geothermal waters from Iwonicz-Zdrój (Kępińska 2011) and Rabka-Zdrój for several years.

Geothermal waters contain large amounts of silica, potassium and microelements such as Li, Sr, B, Br and I. Methods for the extraction, electrolysis and precipitation of sulfate salts from geothermal waters are used in many regions of the world (Gallup 1998, 2007). Boron is a particularly valuable microelement, but for its recovery to be possible, the solution must be highly concentrated, i.e. its content in the solution should amount to several hundred mg/L. Using the process of selective ion exchange to remove boron from water, this element can be precipitated as a result of a reaction with a strong acid (Gallup 1998). Among others, Recepolgu and Beker (1991), Gallup (1998) and Turek et al. (2007, 2008) have presented practical examples of technological solutions allowing boron to be selectively removed from geothermal waters along with the recovery of boric acid from aqueous solutions. Boric acid can be used as a fertiliser, wood preservative, gentle disinfectant and also food preservative. The earliest commercial, geothermal mineral recovery process consisted of boric acid “mining” from steam wells at the Lardarello, Italy field at the beginning of the century (Cataldi et al. 1970; Lund and Freeston 2001).

### Use of low mineral content and fresh waters

Low mineral content and fresh geothermal waters are mostly made available for heating and leisure purposes. In 2010, seven facilities operated in Poland that used geothermal energy for heating purposes (Bujakowski 2010); three of those, fed with low mineral content waters, are heating plants that serve municipal heat distribution networks (Podhale–Bańska Niżna, Mszczonów, Uniejów), and three are plants at leisure complexes that use geothermal water as well as the heat recovered for their own purposes (heating of the “Terma Bukowina Tatrzańska” and “Termy Uniejów” facilities and heating the “Kąpielisko Geotermalne Szymoszkowa” pool in Zakopane).

From 2006 to 2008, six geothermal spas and leisure facilities were constructed. Four of those operate in the Podhale region (“Aqua Park Zakopane”, “Termy Podhalańskie” in Bańska Niżna, the aforementioned “Kąpielisko Geotermalne Szymoszkowa” in Zakopane and “Terma Bukowina Tatrzańska”) and two in central Poland: “Termy Mszczonowskie” and the aforementioned “Termy Uniejów”. Some of those facilities have extended the manner and scope of utilisation of geothermal waters that were hitherto only used for heating purposes. In June 2011, another leisure complex opened in Podhale (“Terma Bania” in Białka Tatrzańska) (Kępińska 2011).

**Fig. 2:**
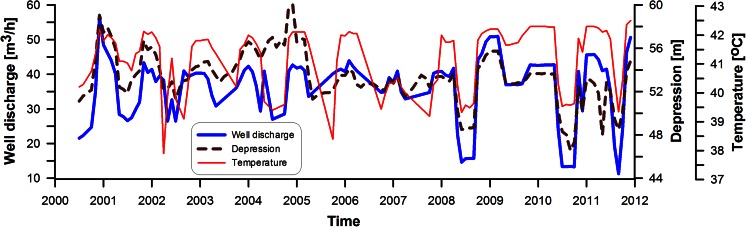
Operating parameters of the Mszczonów IG-1 intake from 2000 to 2011

In Mszczonów (central Poland) (Fig. 1), water with low mineral content (ca. 0.5 g/L) and with an intake temperature of 42 °C has been extracted since 2000 from the Mszczonów IG-1 well—from a Lower Cretaceous horizon composed of sandstones interbedded with mudstone and claystone. These are high-quality Cl-HCO_3_-Na-Ca waters that are fed to the municipal water supply network as drinking water following cooling and simple treatment. The extraction of these waters in an open system with a maximum capacity of 60 m^3^/h (without re-injecting cooled water into the formation) has significantly improved the economic performance of the project and the utilisation of cooled water as drinking water has additionally enhanced the management of ordinary water resources. The flow rate of the water extracted has been steady for 12 years (Fig. 2). A similar manner of utilisation of geothermal waters is envisaged in Poddębice where geothermal water exhibits a mineralisation of ca. 0.4 g/L at intake.

At other geothermal facilities, part or all of the water extracted is dumped, mainly into surface waterways.

Owing to the shortfall of ordinary water in many regions of the world, the desalination and use of geothermal water is increasingly perceived as an advantageous manner of securing high-quality drinking water and water for irrigating crops, while limiting the negative impact resulting from dumping such water into surface waterways (Öner et al. 2011; Gallup 2007; Manenti et al. 2013a, b). Research aimed at assessing the feasibility of using treated geothermal water for drinking purposes has also been undertaken in Poland (Tomaszewska, 2011; Tomaszewska and Bodzek 2013a, b, c). Waters from three different geothermal areas were tested, i.e. from the Podhale basin (Bańska IG-1), Polish Lowlands (Uniejów PIG/AGH-2) and Western Carpathian Mountains (Rabka IG-2). The waters analysed exhibited different mineral contents ranging from 2.5 to 24.4 g/L as well as elevated or high iron, strontium, boron and silica content (Tomaszewska, 2011; Tomaszewska and Bodzek 2013a). It should be stressed that the water desalination process results in the separation of the solution into two streams: (1) the treated water (permeate) stream that exhibits a low content of dissolved substances and (2) retentate (concentrate) that contains separated particles and dissolved substances. In this context, it should be pointed out that the magnitude of the water stream fed to the desalination system should be determined by the manner in which desalinated water is to be used and the possibilities of disposing of the retentate.

### Use of desalinated geothermal water

**Fig. 3:**
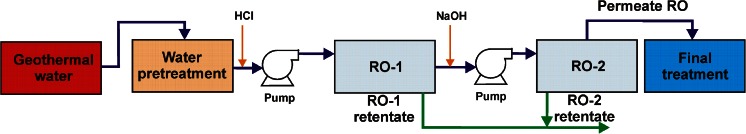
Simplified diagram of the thermal water desalination process at PAS MEERI

Taking into account water salinity (up to 7 g/L) and the increased content of silica, hydrogen sulfide and other sulfides, boron, barium, strontium, ammonium ions, fluorides, bromides and sulfates, a double hybrid setup was selected that combined ultrafiltration (UF) and two independent reverse osmosis systems (RO-1 and RO-2) connected in series (Tomaszewska, 2011; Tomaszewska and Bodzek, 2013a). A schematic diagram of the desalination system is shown in Fig. 3. A detailed description of the water desalination technology together with a technical specification of the UF and RO membranes and the description of the tests conducted was presented in Tomaszewska and Bodzek (2013a, b, c).

Taking into account the low pressure applied in the reverse osmosis process (1.1 MPa), a relatively high removal rate was received—96–97 % with respect to conductivity and 94 % with respect to SiO_2_, 92 % for fluoride and not less than 84 % for arsenic (Tomaszewska and Bodzek, 2013a). The utilisation of a two-stage RO system with water reaction adjustment before the RO-2 stage was necessary owing to high boron ion concentrations in the water fed to the facility. Ultimately, the retention ratio for this element ranged from 96 to 97 %. A high rejection ratio of radionuclides was also obtained, ranging from 70.7 to 99.6 % (Tomaszewska and Bodzek 2013c). A comparison of physical and chemical properties of desalinated water against the standards applicable to drinking water has been presented in Table 1.

**Table 1:** A comparison of physical and chemical properties of desalinated water against the standards applicable to drinking water

Parameters	Bańska IG-1	Uniejów PIG/AGH-2	Rabka IG-2^*^	National Drinking Water Standard
TDS, mg/L	181.5	291.6	2,588.0	−
Total hardness, mg CaCO_3_/L	60	60	25.6	60–500
Carbonate hardness, mg CaCO_3_/L	60	60	25.6	−
Conductivity, mS/cm	0.417	1 887	4,500	2,500
SiO_2_, mg/L	0.198	0,31	2.15	−
Na, mg/L	40.88	151.8	575.1	200
K, mg/L	0.83	1.76	8.19	−
Ca, mg/L	55	59	7.11	−
Mg, mg/L	10	11	1.905	30–125
Cl, mg/L	7.6	11.2	1,294.0	250
SO_4_, mg/L	6.4	<3.0	<3.0	250
As, mg/L	0.001	<0.005	0.006	0.010
B, mg/L	0.24	0.159	32.98	1.0
Cr, mg/L	<0.005	<0v005	0.095	0.050
Cd, mg/L	<0.005	<0.0005	<0.0005	0.005
Ni, mg/L	<0.001	<0.005	<0.005	0.020
Pb, mg/L	0.0005	<0.0005	<0.0005	0.025^a^/0.010^b^
Hg, mg/L	<0,0,001	<0.0001	<0.0001	0.001
Al, mg/L	0.005	<0.01	0.02	0.200
Mn, mg/L	<0.003	<0.005	0.013	0.050
Fe, mg/L	0.013	0.03	0.037	0.200
F, mg/L	0.137	0.078	0.104	1.5
Sr, mg/L	0.006	<0.2	0.907	−

^a^Shall apply until 31 December 2012

^b^Shall apply from 1 January 2013

*Desalination of water from Rabka IG-1 well requires a transmembrane pressure higher than 1.1 MPa

As a result of desalination of waters with mineral content of up to 3 g/L, high-quality water that met the requirements set forth in the PN-85/C-04601 Polish standard was obtained, which could be used in municipal central heating networks (secondary circulation).

### Ordinary water shortfall

Water is the basis for life and culture. In addition to the availability of water, its quality has become a major issue in industrialised areas and in developing countries as well (Frimmel 2003). In certain circumstances, the use of desalinated geothermal water may constitute an alternative enabling local needs to be met. Drinking water shortfalls affect an increasing number of countries (Frimmel 2003; Crosa et al. 2006) including some areas in Poland.

**Fig. 4:**
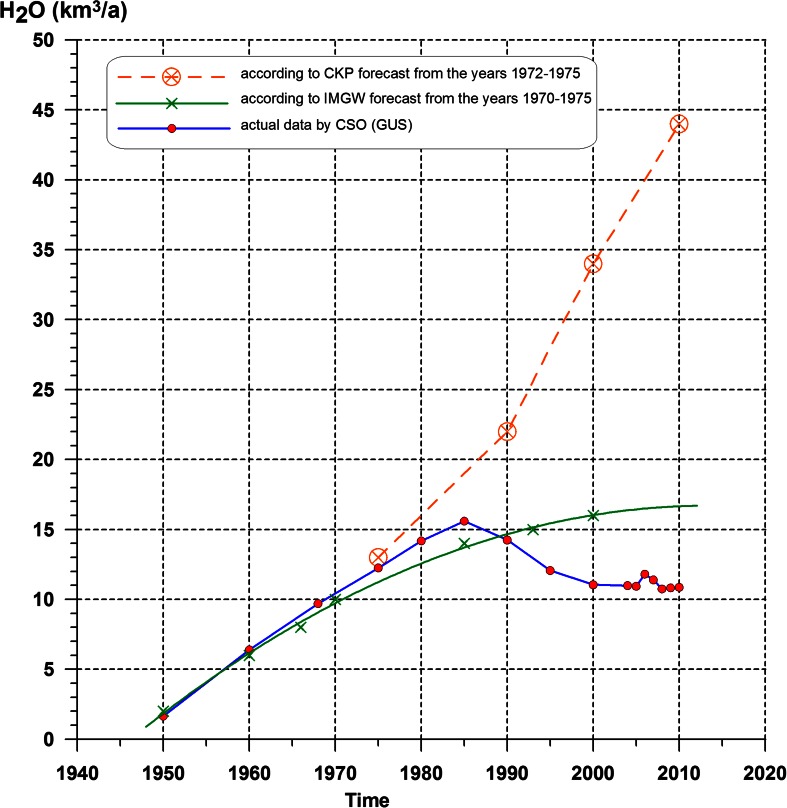
Water consumption in Poland, 1950–2010 (based on Szczepański 2008, supplemented)

Despite limited water resources in Poland, its consumption rose sharply after WWII, and the forecasts developed in the 1970s significantly exceeded the capacity to meet the increasing needs (Fig. 4). While the average available resources that could be used annually were at the level of 22.5 km^3^/a, forecasts reached 34 km^3^/a (in 2000) and even 44 km^3^/a (in 2010), which was practically unattainable from the point of view of rational water management. Maximum capacity is estimated at 16 km^3^/a provided that storage reservoirs are constructed (Szczepański 2008).

Variations in the intensity of water circulation lead to random changes in water resources. In recent decades, certain trends have become noticeable with respect to average and extreme river flow rates, water levels in rivers and lakes and groundwater levels (Kundzewicz and Kowalczak 2008; Witczak et al. 2007). Average water resources in Poland amount to around one-third of the European average at 1,580 m^3^/person/year, while in dry years, they do not exceed 1,000–1,100 m^3^/person/year. In more sophisticated classifications, temporal and spatial variability of water resources is taken into account together with the country’s economic situation in GDP terms, which affects the ability to adapt to difficulties with managing water resources (Kowalczak 2008). In Poland, areas with significant shortfalls of both surface waters and groundwater have been observed for years now. Paczyński and Sadurski (2007) divide such areas into three types in terms of their origins: (1) the presence of threats to the sustainable use of surface and groundwater resources, (2) increased consumption related to a group of heavily exploited groundwater intakes within the resource area in question, and (3) the absence of intakes that could be used for water supply purposes. The last case can be observed, *inter alia*, in the Podhale trough (Bańska Niżna, Szaflary) where groundwater intake is dispersed and limited and only 20 % of households (i.e. ca. 500 houses) are connected to the water supply network.

### Use of the concentrate obtained during geothermal water desalination

The characteristics of the waste stream depend on the quality of the feed water, the quality of the produced water (depend of recovery varies level), the pre-treatment method (added chemicals) and the cleaning procedures used.

The concentrate must be disposed of in an appropriate manner; it should be sold where possible. Owing to its salinity and environmental considerations, the concentrate must not be discharged into surface waters. To enable the utilisation of the concentrate following the RO process, it is advisable to limit the amount of chemicals used.

The examination of physical and chemical properties of the concentrate obtained during research involving water from the Bańska IG-1 and Uniejów PIG/AGH-2 wells demonstrated (Tomaszewska and Pająk, 2012) that the concentrate may be widely used, including as an alternative balneological product. The microelements present in geothermal water such as arsenic, barium, boron, heavy metals etc. may restrict the possibilities in some cases.

A comparison of analysis results of the retentate (Table 2) obtained as a result of the desalination of water from the wells under consideration with the aforementioned guidelines demonstrates that the concentrate meets the expected parameters for waters used externally. The content of dissolved substances in the concentrated solution obtained as a result of desalinating water from the wells covered by the study significantly exceeds the concentrations found in “raw” geothermal water (Table 2). The TDS of the concentrate obtained as a result of desalinating the water from the Bańska IG-1 intake exhibits a mineral content of 8.78 g/L, with an elevated concentration of specific substances that determine the therapeutic/balneological properties of water: metasilicic acid (262.99 mg/L), fluoride ions (5.92 mg/L) and iodide ions (2.47 mg/L). As a result of the solution having been concentrated, the boron ion content of water increased to 22.86 mg/L. The TDS of the concentrate obtained as a result of desalinating the water from the Uniejów PG/AGH-2 well exhibits a mineral content of 17.506 g/L, with an elevated concentration of metasilicic acid (106.03 mg/L), and boron content of ca. 13.8 mg/L (Table 2). In these both cases, no substances were found that would prevent the utilisation of the concentrate for external use. It should be emphasised that owing to its temperature (ca. 30 °C), the concentrate can still be used as geothermal water.

**Table 2:** Comparison of concentrate analysis results with the highest admissible concentrations of ingredients that are undesirable in excessive amounts and toxic ingredients in therapeutic waters pursuant to the Regulation of the Minister of Health (2006)

Parameter	Bańska IG-1 concentrate	Uniejów PIG/AGH-2 concentrate	The highest admissible concentrations		
			Drinking cure	Inhalation	Bathing
TDS, mg/L	8785.1	17506.0	−	−	−
Total hardness, mg CaCO_3_/L	2,115	996.4	−	−	−
Carbonate hardness, mg CaCO_3_/L	237.8	153	−	−	−
Conductivity, mS/cm	11.369	35.3	−	−	−
SiO_2_, mg/L H_2_SiO_3_, mg/L	202.32 262.99	81.56 106.03	−	−	−
Na, mg/L	1,794	5,724	−	−	−
K, mg/L	145.13	60.85	−	−	−
Ca, mg/L	645.1	313.66	−	−	−
Mg, mg/L	122.83	51.98	−	−	−
Cl, mg/L	2433.4	9,334	−	−	−
SO_4_, mg/L	2818.72	316.2	−	−	−
As, mg/L	0.0165	0.070	−	−	−
B, mg/L	22.86	13.85	5.0	30.0	−
Cr, mg/L	0.081	0.437	0.01	0.01	−
Cd, mg/L	<0.0005	<0.0005	0.003	0.003	−
Ni, mg/L	0.079	0.006	0.03	0.03	−
Pb, mg/L	0.0011	<0.0005	0.01	0.01	−
Hg, mg/L	0.0009	<0.0001	0.001	0.001	−
Al, mg/L	<0.010	0.018	0.1	0.1	−
Mn, mg/L	0.434	0.169	−	−	−
Fe, mg/L	28.73	0.381	−	−	−
F, mg/L	5.92	0.06	−	−	−
Sr, mg/L	18.64	12.502	−	−	−
Li, mg/L	3.53	0.688	−	−	−
Ba, mg/L	0.189	0.385	1.0	10.0	−
Zn, mg/L	0.068	<0.01	−	−	−
Cu, mg/L	0.019	0.056	−	−	−
Co, mg/L	0.0014	<0.0005	−	−	−
PO_4_, mg/L	0.662	0.2215	−	−	−
I, mg/L	2.47	1.4	−	−	−

Summing up the results related to spent geothermal water desalination obtained to date, it may be stated that following the treatment presented (Tomaszewska 2011; Tomaszewska and Bodzek 2013a), the water may be used to meet local needs, while the concentrate may be used for balneological and leisure purposes.

## Summary

Poland has relatively favourable geothermal conditions that are comparable with most European countries. In accordance with legal requirements and also customarily, before projects that aim to extract geothermal waters are planned, detailed analyses of geological, hydrogeological and geothermal conditions in the location in question and in its vicinity are conducted, and the probability of obtaining assumed reservoir and operational parameters at the projected drilling site is evaluated; financial expenditure is estimated, and project financial feasibility is analysed. Knowledge of geological features and underground conditions at the location in question is a key from the point of view of making decisions concerning exploration drilling. At the project planning phase, an equally important question that requires detailed analysis is the evaluation of the directions and manner of utilisation of spent water. A model procedure should take the following aspects into account: ensuring that geothermal energy resources are renewable, enabling safe long-term reservoir operation and ensuring that activities are both cost-effective and environmentally sound. Therefore, closed systems are the best and safest from the point of view of the geothermal water reservoir. However, owing to the still high cost of drilling and problems related to the corrosion and clogging of absorption wells, this manner of water utilisation is limited to just a few cases.

Alternative solutions such as using cooled water directly for drinking or household purposes are advantageous ones in certain cases, which are confirmed by the activities of Geotermia Mazowiecka S.A. The implementation of desalination processes involves certain costs which are determined by factors such as the quality of raw water, desalination facility size, facility location, the manner in which the concentrate is disposed of, the quality and skills of the workforce, the cost and type of energy used and the type of technology used. Outcomes may differ depending on local economic and environmental conditions. Membrane-based water desalination technologies and also hybrid systems are widely used to produce drinking and household water in many regions of the world. They are also a technological and economic alternative supported by renewable energy (solar, wind or geothermal energy). They can and should be used in Poland as well.

Geothermal waters are a source of clean energy as well as a valuable product with therapeutic, balneotherapeutic and recreational properties. They should be used in a rational manner—in energy and economic as well as environmental terms.
